# Implementation of Stereotactic Accelerated Partial Breast Irradiation Using Cyber-Knife – Technical Considerations and Early Experiences of a Phase II Clinical Study

**DOI:** 10.1007/s12253-020-00821-3

**Published:** 2020-05-29

**Authors:** Norbert Mészáros, Viktor Smanykó, Tibor Major, Gábor Stelczer, Levente Jánváry, Eszter Kovács, Bahéri Mária, Zoltán Zaka, Dávid Pukancsik, Zoltán Takácsi-Nagy, Csaba Polgár

**Affiliations:** 1 Center of Radiotherapy, National Institute of Oncology, Ráth György u. 7-9, Budapest, H-1122 Hungary; 2 Department of Oncology, Semmelweis University, Faculty of Medicine, Budapest, Hungary; 3 Department of Radiology, National Institute of Oncology, Budapest, Hungary; 4 Department of Breast and Sarcoma Surgery, National Institute of Oncology, Budapest, Hungary

**Keywords:** Breast cancer, APBI, SBRT, Cyber-knife, Phase II

## Abstract

To report the implementation, dosimetric results of and early experiences with stereotactic accelerated partial breast irradiation (SAPBI) following breast conserving surgery (BCS) for postmenopausal low-risk St I-II invasive breast cancer (IBC) patients. Between November 2018 and August 2019, 27 patients were registered in our phase II prospective study. SAPBI was performed with Cyber-Knife (CK) M6 machine, in 4 daily fractions of 6.25 Gy to a total dose of 25 Gy. Respiratory movements were followed with implanted gold markers and Synchrony system. Corrections for patient displacement and respiratory movement during treatment were performed with the robotic arm. Early side effects, cosmetic results, and dosimetric parameters were assessed. The average volume of the surgical cavity, clinical target volume (CTV), and planning target volume (PTV_EVAL) were 8.1 cm^3^ (range: 1.75–27.3 cm^3^), 55.3 cm^3^ (range: 26.2–103.5 cm^3^), and 75.7 cm^3^ (range: 40–135.4 cm^3^), respectively. The mean value of the PTV_eval/whole breast volume ratio was 0.09 (range: 0.04–0.19). No grade 2 or worst acute side-effect was detected. Grade 1 (G1) erythema occurred in 6 (22.2%) patients, while G1 oedema was reported by 3 (11.1%) cases. G1 pain was observed in 1 (3.4%) patient. Cosmetic result were excellent in 17 (62.9%) and good in 10 (37.1%) patients. SAPBI with CK is a suitable and practicable technique for the delivery of APBI after BCS for low-risk, St. I-II. IBC. Our early findings are encouraging, CK-SAPBI performed with four daily fractions is convenient and perfectly tolerated by the patients.

## Introduction

The most frequent malignancy among women in industrialized countries is breast cancer (BC). According the WHO 627,000 women died from BC worldwide – which is almost 15% of all cancer deaths among women in 2018 (https://www.who.int/cancer/prevention/diagnosis-screening/breast-cancer/en/). Due to the introduction of mammography screening and more effective oncological treatments, BC mortality shows a downward trend. Several prospective randomized studies demonstrated that in early-stage BC patients whole breast irradiation (WBI) following breast conserving surgery (BCS) not only reduces the proportion of local recurrences (LR) in the ipsilateral breast, but also improves overall survival [1–3]. The rationale for WBI is the eradication of residual microscopic disease after lumpectomy. However, WBI exposes the skin, lung, chest wall, and heart to high doses of radiation. The theory of accelerated partial breast irradiation (APBI) stems from two reasons. First, the predominance of LR’s after BCS are near to the original primary tumour site. According to the pathological studies of Holland et al., multicentricity occurred in a significant proportion of BC’s, therefore, WBI was the standard treatment after BCS [4]. Later, other pathological studies demonstrated that in a unicentric, unifocal tumour (without extensive intraductal component or invasive lobular pathological subtype), microscopic tumour cells can be found only in 2–4% at >1.5–2 cm distance from the index tumour, and following WBI the ipsilateral breast recurrence rate was almost equivalent to the rate of contralateral carcinomas [5, 6]. Second, even in developed countries, radiotherapy units are not available in all cities, therefore, the five-days-a-week occasions for 3 to 7 weeks treatment schedule may expect patients to omit work and can lead to other daily routine severances. Almost 20% of patients with early-stage IBC and treated with BCS choose to forgo the radiation therapy in the United States [7]. To minimalize the treatment schedule from 3 to 7 weeks to 1 week or less is another advantage of APBI. These pathological findings and the clinical eagerness to abbreviate the course of breast radiotherapy and decrease the toxicity of normal tissues led to the investigation of efficacy and safety of APBI in prospective clinical trials [8–25]. In these early studies, the inappropriate patient selection criteria, quality control, and target definition led to very high (25–37%) local relapse rates after 6–8 years [8, 9]. In later studies, more favourable experiences accumulated using APBI with interstitial brachytherapy (iBT), three-dimensional conformal external beam radiotherapy (3D-CRT), and image-guided intensity-modulated radiotherapy (IG-IMRT) in correctly selected early-stage BC patients [10–25]. Stereotactic treatment approaches are emerging and are widely accepted in early stage lung cancer, prostate cancer, or bone and brain metastasis patients [26–29]. However, to date, only a few phase I/II studies were published with CyberKnife-based stereotactic APBI (CK-SAPBI) [30–36].

In 2018, we initiated a phase II clinical study to test the effectiveness and usefulness of CK-SAPBI with a four-fraction schedule. SAPBI adopting real-time tracking, respiratory motion administration, and submillimetre efficiency offers improvements in partial breast irradiation enabling great dose conformality to target region [31, 36]. We report the dosimetric parameters and the early clinical outcomes of this prospective phase II clinical study using CK-SAPBI.

## Patients and Methods

**Fig. 1:**
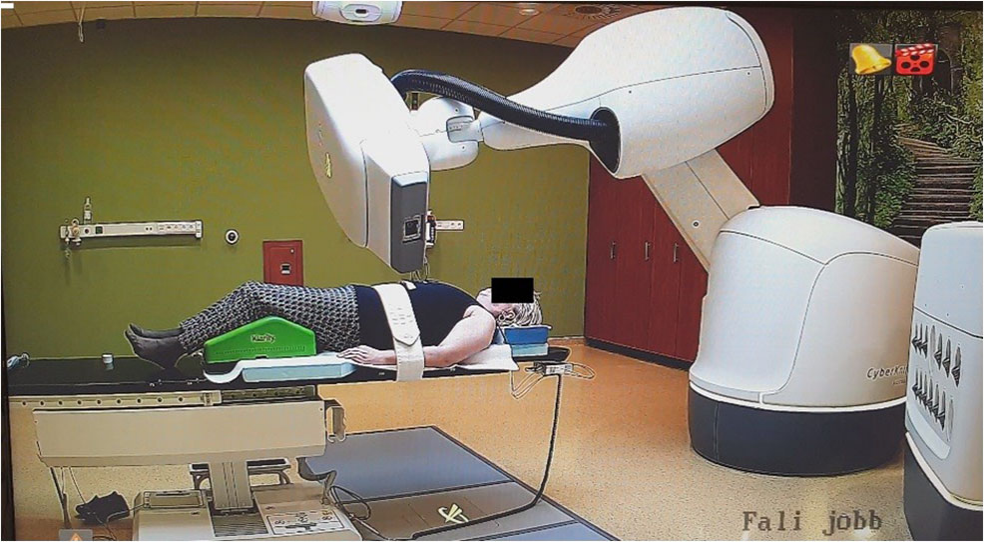
S-APBI with M6 Cyberknife machine

**Fig. 2:**
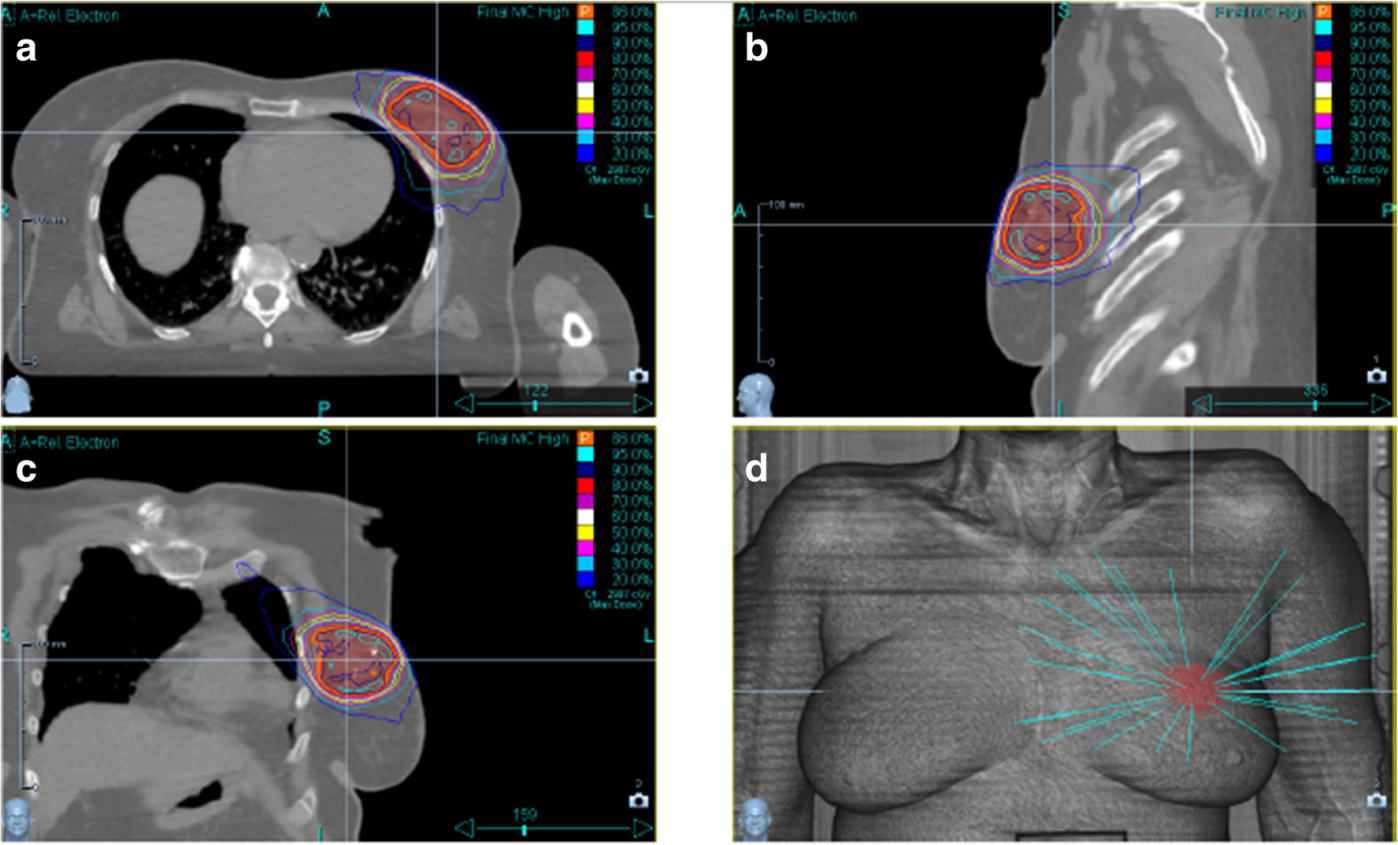
Cyber-Knife treatment planning images with the dose of 4 × 6.25 Gy. **a-c** isodose lines in three different planes. **d** illustration of pencil beam trajectories

Between 2018 November and 2019 August, 27 early-stage BC patients were treated with CK-SAPBI. Patient eligibility criteria were the same as in our previous APBI trials, and were reported in detail in our previous publications [19, 20, 22, 23]. Briefly, patients were eligible for CK-SAPBI if they were 50 years old or older, with invasive tumour up to a diameter of 3 cm, with negative axillary lymph node status (pN0) and with clear surgical margins by at least 2 mm. We omitted patients if they had multiple or multifocal invasive tumour histology subtype Paget-disease, bilateral breast cancer, preliminary history of BC or additional malignant disease within 5 years. Tumour and patient characteristics are presented in Table 1. To identify the excision cavity and to guide optimal fiducial placement, CT scanning of the operated breast was performed with a 5 mm slice thickness. Only patients with good identifiable cavity were eligible for the study (visibility score (CVS) of 3–5) [37]. Prior to the treatment, three to four gold fiducials were placed near the surgical bed with ultrasound guidance by a certified radiologist together with the treating radiation oncologist, in local anaesthesia. For optimal tracking, the maximum and minimum distances between fiducial markers were less than 10 cm and more than 2 cm, and the markers were placed at least 1–2 cm away from the seroma cavity, as recommended by Seiler et al. [38]. One week after gold marker placement, a new simulation CT was performed in a supine position with both arms next to the body with 1.25 mm axial slice spacing. For delineating the target volumes and organs at risks, the GEC-ESTRO Breast Working Group recommendations were applied [39]. The surgical bed included the surgical clips were determined as the tumour bed. The clinical target volume (CTV) was defined as the tumour bed+ (20 mm minus the free surgical margins (in mm)) in six directions. The CTV margin was confined at the breast parenchyma/pectoral muscle interface and to 5 mm beneath the skin surface and. A uniform, three-dimensional 2 mm margin was added to the CTV to attain the planning target volume (PTV). For dosimetric reporting, the PTV_EVAL was accomplished from the PTV limiting the PTV except the first 5 mm of tissue from surface and any lung tissue [15]. Daily localization and fiducial tracking were accomplished with the Synchrony Respiratory Tracking System (Cyberknife, Accuray, Sunnyvale, CA). Light-emitting diodes (LED’s) were placed on the chest wall of the patients and were tracked by an optical camera. Fifteen orthogonal x-ray image pairs were acquired in different phases of the breathing cycle. The fiducials were checked on each image pair. A predictive correlation model was created between tumour bed positions – represented by the fiducials – and the chest wall position. During beam delivery, the radiation fields were always pointing at the position of the PTV, according to the information obtained from the optical camera signal. A minimum of 3 fiducial markers were tracked at all times to avoid rotational errors. The treatments were performed on a CyberKnife M6 machine with MLC and step-and-shoot IMRT technique, with 57 segments on average (range: 34–76), and with a dose of 4 × 6.25 Gy (total dose: 25 Gy) on four consecutive days (Figs. 1 and 2). Our equivalent dose calculations are listed in Table 2, compared to other fractionations (10 × 3.4 Gy and 7 × 4.3 Gy) frequently used by others in routine clinical practice. Radiotherapy was started within 12 weeks after surgery for all 27 patients. Hormone therapy was applied to all patients, 26 patients (96.6%) received aromatase inhibitors or goserelin acetate and tamoxifen in 1 case (3.4%). Within 7–14 days after the completion of CK-SAPBI, the acute side effects were recorded according to the Radiation Therapy Oncology Group/European Society for Therapeutic Radiology and Oncology (RTOG/EORTC) scoring system [40].

**Table 1:** Patient, tumor and adjuvant treatment characteristics

Characteristics	n (%)^a^
Mean age (range)	65 ys. (50–77)
Age groups (years)	
50–60	5 (18.5%)
61–70	17 (63%)
> 70	5 (18.5%)
Postmenopausal	26 (96.3%)
Breast cup size	
A	7 (25.9%)
B	6 (22.2%)
C	12 (44.4%)
D, D+	2 (7.5%)
Laterality	
Right	14 (51.8%)
Left	13 (48.2%)
Tumour location (quadrant)	
Upper-outer	15 (55.6%)
Lower-outer	4 (14.7%)
Upper-inner	5 (18.6%)
Lower-inner	1 (3.6%)
Central	2 (7.5%)
Pathological tumour size (mm)	
≤ 5	0 (0%)
> 5–10	12 (44.4%)
> 10–20	13 (48.2%)
>20–30	2 (7.4%)
Median (mm)	10
Pathological nodal status	
pN0 (SLNB)	27 (100%)
pN0 (ALND)	0 (0%)
Free surgical margins (mm)	
≥ 2–5	12 (44.4%)
> 5–10	15 (55.6%)
> 10	0 (0%)
Histologic type	
Ductal invasive	27 (100%)
Histologic grade	
1	13 (48.2%)
2	13 (48.2%)
3	1 (3.6%)
Hormone receptor status	
ER and PR +	26 (96.4%)
ER +, PR -	1 (3.6%)
ER -, PR +	0 (0%)
ER and PR -	0 (0%)
Endocrine therapy	
Yes	27 (100%)
No	0 (0%)
Chemotherapy	
Yes	0 (0%)
No	27 (100%)

*ER* estrogen receptor, *PR* progesterone

^a^receptor Data are n (%) if not otherwise specified

**Table 2:** Calculations of equivalent doses in different fractionations with using different α/β ratio

	Total dose	BED Gy_4_	BED Gy_10_	BED Gy_2_	EQD2 Gy_4_
10 × 3.4Gy	34	63	46	92	42
7 × 4.3Gy	30.1	63	43	96	41.6
4 × 6.25Gy	25	64	40.6	103	42.7

Biological equivalent dose (BED) and equivalent dose in 2 Gy per fraction (EQD2) for tumour control and for normal tissue toxicity at each dose level

The study protocol was assessed and approved by the national ethics committee, and before recruitment all women provided written informed consent.

## Results

All patients received the planned four fractions of CK-SAPBI with the predefined study-specific dose constraints. No protocol violation arose. At least three fiducials were tracked in all cases. The mean size of the primary tumour was 11 mm (range: 6–21 mm). The average volume of the surgical cavity, CTV and PTV_EVAL was 8.1 cm^3^ (range: 1.75–27.3 cm^3^), 55.3 cm^3^ (range: 26.2–103.5 cm^3^), and 75.7 cm^3^ (range: 40–135.4 cm^3^), respectively. The mean value of the PTV_eval/whole breast volume ratio was 0.09 (range: 0.04–0.19). Dose-volume parameters are listed in Table 3. The average values of V100 for CTV and PTV_EVAL were 99.4% and 97.5%, respectively. The mean Dmax value for PTV_EVAL was 116.5%. The mean V_100_, V_75_ and V_50_ of ipsilateral breast-PTV was 0.73% (range: 0.3–1.4), 4.8% (range: 2–8.5%) and 10.8% (range: 5–18.9%) respectively. The mean ipsilateral and contralateral lung dose (MLD) and D_10%_ was 131 cGy (range: 33–205 cGy) and 328 cGy (range: 68–524 cGy), 12 cGy (range: 3–44 cGy) and 29 cGy (range: 8–131 cGy), respectively. The mean heart dose (MHD) and D_0,04cm3 heart_ was 89 cGy (range: 31–173 cGy) and 568 cGy (range: 216–1094 cGy) for left-sided, and 34 cGy (range: 13–84 cGy) and 219 cGy (range: 90–436 cGy) for right-sided lesions, respectively. The mean D_0,01cm_^3^, and mean D _0,04cm3_ skin and rib doses was 2380 cGy (range: 1074–2866 cGy), 2338 cGy (range: 1021–2839 cGy) and 2317 cGy (range: 978–2766), 2243 cGy (range: 952–2722 cGy) respectively. A typical treatment plan are shown in Fugure 2.

**Table 3:** Dose volumes parameters of organs at risks

Organs at risks		
	Mean	Median (range)
Ipsilateral non-target breast (%)		
V 100%	0.7	0.7 (0.3–1.4)
V 75%	4.8	4.3 (2.0–8.5)
V 50%	10.7	9.5 (5–18.9)
Contralateral breast		
D 0.04 cm3 (cGy)	50.4	39 (13–172)
Mean dose (cGy)		
Heart (left sided lesion)		
D 0.04 cm3 (cGy)	568	477 (216–1094)
Mean dose (cGy)	89	83 (31–173)
Heart (right sided lesion)		
D 0.04 cm3 (cGy)	219	201 (90–436)
Mean dose (cGy)	34	33 (13–84)
Ipsilateral lung		
D 10% (cGy)	328.3	337 (68–524)
Mean dose (cGy)	131.6	129 (33–205)
Contralateral lung		
D 5% (cGy)	38.8	31 (16–164)
Mean dose (cGy)	12.1	10 (3–44)
Skin		
D 0.01 cm3 (cGy)	2380.1	2674 (1074–2866)
Rib		
D 0.01 cm3 (cGy)	2317.4	2475 (978–2766)

At a median follow-up of 12 months (range: 8–17 months), no loco-regional recurrence or distant metastasis occurred, and all patients are alive. Grade 2 or worse early side-effect wasn’t detected. Grade 1 (G1) erythema occurred in 6 (22.2%) patients, while G1 oedema, was reported in 3 (11.1%) cases. G1 pain was observed by 1 (3.4%) patient. The cosmetic outcome was excellent in 17 (62.9%), and good in 10 (30.1%) patients. Cosmetic results and early side effects are listed in Table 4. The average daily treatment time was 43 min (range: 33–68 min) door-to-door.

**Table 4:** Early radiation side effects and cosmetic results

	Grade 0	Grade 1	Grade 2	Grade 3–4
Early side effect				
Skin	21 (77.8%)	6 (22.2%)	0 (0%)	0 (0%)
Breast parenchyma	24 (89.9%)	3 (11.1%)	0 (0%)	0 (0%)
Pain	26 (96.6%)	1 (3.4%)	0 (0%)	0 (0%)
	Excellent	Good	Fair	Poor
Cosmetic result				
Rated by physicians	17 (61.9%)	10 (30.1%)	0 (0%)	0 (0%)
Rated by patients	16 (59.3%)	11 (40.7%)	0 (0%)	0 (0%)

## Discussion

At our institute, between November 2018 and August 2019, 27 patients were treated with SAPBI using a Cyber-Knife M6 machine, with four daily fractions. According to our early experience, no patients have recurrence or fair/poor cosmesis.

APBI with appropriate patient selection and quality control became a widely accepted treatment option for the treatment of early-stage BC in highly qualified radiotherapy centres [10–25]. In the GEC-ESTRO Breast Cancer Working Group, multicentric randomised study iBT still prove the efficacy, feasibility, and quality of life after a 5-year follow-up. But brachytherapy is invasive and needs a highly qualified staff to perform [10–12]. In 2019, Vicini et al. presented the 10-year results of the NSABP B-39/RTOG 0413 phase III APBI study in the Lancet [41]. The 10-year cumulative incidence of IBTR between APBI and WBI was only 0.7% (4.6% vs 3.9%) with a hazard ratio (HR) of 1.22 (90%CI 0.94–1.58), this difference in IBTR was less than 1% at 10 years, suggesting that APBI is an acceptable option for a proportion of women who undergo BCS. There were no statistically significant differences in late G3–5 toxicities. In the same issue of the Lancet, the long-term results of the RAPID 3D-CRT APBI trial were also presented by Whelan et al. [42]. After 8.6 years of median follow-up, the LR rate was 2.8% in the WBI, and 3% in the PBI arm, but the difference was not statistically significant [HR = 1.27 (90%CI, 0.84–1.91)]. Grade 2 and grade 3 late side effects were 28% and 4% in PBI and 12% and 1% in the WBI arm, the 7-year fair and poor cosmetic results were 31% vs 15%, respectively. The five-year results of the Italian prospective randomized study have presented that the 5-year local tumour control was equivalent with APBI or WBI, 1.5% local recurrence rate in both arms [43]. Concerning acute side effects, the APBI group showed significantly better results in any grade (*p* = 0.0001) and grade 2 or higher (p = 0.0001) cases. APBI with 3D-CRT or IG-IMRT are non-invasive techniques in contrast to iBT.

Recently, CK has emerged as a possible alternative to conventional APBI techniques. CyberKnife offers advantages of iBT and external beam APBI. The dose profile of CK is comparable to brachytherapy because of the steep dose gradient with small PTV volumes, and it is a non-invasive method. The CK respiratory tracking system allows treatment to be delivered while also considering the patient’s breath motions. Many publications have noted higher rates of late toxicity and/or poorer cosmetic results following APBI delivered with 3D-CRT or IMRT compared to WBI [14, 18, 37, 44, 45]. Hepel et al. [37] reported 8.3% rate of grade 3–4 subcutaneous fibrosis after a median follow-up of 15 months. Shah et al. [14] also published a 7.5% rate of grade 3–4 fibrosis after 5 years. In these studies, the higher rate of late toxicity was probably due to the voluminous irradiated target, since the PTV values were in the range of 175 to 330 cm^3^. To minimize the amount of the treated volume, we performed real-time image guidance during each fraction. Using the Synchrony system, the CTV-PTV margins could be decreased to 2 mm obtain a limited mean target volume (PTV_EVAL) of 75.7 cm^3^. In contrast, in our previous APBI study using IG-IMRT technique without real-time tracking and using a 5 mm CTV-PTV margin, the mean treatment volume (PTV_EVAL) was 152.6 cm^3^ which is twice the volume obtained in this study using CK-SAPBI [22].

A very limited number of phase I or II prospective study have been published with CK-SAPBI [30–36]. One of the earliest studies published by Vermuelen et al. in 2011 reported 46 patients treated with CK-SAPBI [31]. The first two patients were treated with 5 fractions of 5 Gy each, thereafter the patients received 10 times 3.4 Gy to the PTV, recommended to the 65–75% isodose-line. After 12 patients, the prescribed dose was increased to 10 times 3.6 Gy. The mean percent isodose prescription line was 70% (range, 65–76%) to the PTV, the mean PTV was 114 cm^3^ (range, 39–241 cm^3^). After a mean follow-up of 31 months (range, 6–57 months), no BC recurrence was recorded. Very mild toxicities occurred, 100% of the patients had good-excellent cosmesis. Lozza et al. published a pilot study in 2018, they treated 20 patients with CK-SAPBI using the Iris collimator [32]. The total dose was 30 Gy in 5 consecutive fractions and were prescribed to the isodose line include 95% of the PTV. After a 2-year median follow-up, no recurrence occurred, a mean 88.1 cm^3^ PTV was treated with mild acute and late side effects, and more than 80% of cases ended with excellent cosmetic results. The total patient treatment time comprised patient set-up was around 60 min (range: 35–120 min). In 2016, 10 patients received CK-SAPBI in Georgetown University Hospital [30]. A 5 mm safety margin was added to CTV to generate PTV, and they use the same treatment schedule as previously described (30 Gy in five fractions). The mean prescription isodose line was 80% to the PTV, and the mean treated PTV was 70 cm^3^, 100% of the PTV received the prescription dose (PTV30). After the treatments mild toxicity was reported. At a median follow-up of 1.3 years, no breast events have been documented, and all patients had excellent/good cosmetic results.

There are some potential disadvantages of CK-SAPBI. The impalement of the fiducials is an invasive intervention. Fiducial movement can appear after placement, therefore our practice is to wait at least 1 week following fiducial implantation. CK-SAPBI daily treatment times are lengthened compared to another external beam APBI 10–15 min treatments. In our study, the mean treatment time was 43 min (range: 33–68 min). We observed a learning curve meaning a continuously decreasing door to door treatment time as our experience has grown. The smaller treatment volume and less toxicities are the trade-offs for the relatively longer treatment time.

## Conclusions

SAPBI with CK is a suitable and practicable technique for the delivery of APBI after BCS for low-risk, St. I-II. IBC. Our early findings are encouraging, CK-SAPBI delivered with four daily fractions is convenient and perfectly tolerated by the patients. Longer follow-up and a higher number of patients are needed to validate the late toxicity and tumour control results.
